# Synthesis and Characterization of Dimeric Artesunate Glycerol Monocaprylate Conjugate and Formulation of Nanoemulsion Preconcentrate

**DOI:** 10.3390/molecules28135208

**Published:** 2023-07-04

**Authors:** Rana Hore, Nazmul Hasan, Karsten Mäder, Jörg Kressler

**Affiliations:** 1 Department of Chemistry, Martin Luther University Halle-Wittenberg, Von-Danckelmann-Platz 4, D-06099 Halle (Saale), Germany; 2 Institute of Pharmacy, Martin Luther University Halle-Wittenberg, D-06099 Halle (Saale), Germany

**Keywords:** malaria, dimerization, artesunate, glycerol monocaprylate, dimeric artesunate glycerol monocaprylate conjugate, nanoemulsion preconcentrate

## Abstract

Malaria is one of the major life-threatening health problems worldwide. Artesunate is the most potent antimalarial drug to combat severe malaria. However, development of drug resistance, short plasma half-life, and poor bioavailability limit the efficacy of this drug. Here, we applied the dimerization concept to synthesize dimeric artesunate glycerol monocaprylate conjugate (D-AS-GC) by conjugating artesunate (AS) with glycerol monocaprylate (GC) via esterification reaction. D-AS-GC conjugate, AS, and GC were well characterized by ^1^H NMR, attached proton test (APT) ^13^C NMR and 2D NMR spectroscopy. D-AS-GC conjugate was further analyzed by ESI-TOF MS. Finally, a series of nanoemulsion preconcentrate (F1–F6) of D-AS-GC was prepared by mixing different ratios of oil and surfactant/cosurfactant and evaluated after dilution with an aqueous phase. The optimized formulation (F6) exhibits a clear nanoemulsion and the hydrodynamic diameter of the dispersed phase was determined by DLS and DOSY NMR spectroscopy. The morphology of the nanoemulsion droplets of F6 was investigated by AFM, which revealed the formation of tiny nanoemulsion droplets on a hydrophilic mica substrate. Moreover, using a less polar silicon wafer led to the formation of larger droplets with a spherical core shell-like structure. Overall, the rational design of the dimeric artesunate-based nanoemulsion preconcentrate could potentially be used in more efficient drug delivery systems.

## 1. Introduction

One of the significant challenges to the public health sector, especially in developing nations, is the prevalence of the malaria disease. Nearly half of the world’s population is susceptible to the risk of malaria infection, establishing it as one of the most prominent health issues in humans [1]. According to the World Health Organization (WHO), approximately 241 million malaria incidences and 627,000 fatalities were reported worldwide in 2020 [2]. Antimalarial drugs are the main means to treat and fight malaria. However, the development and spread of resistance to the majority of traditional antimalarial drugs, such as quinoline derivatives and antifolates, which have been the mainstay over years, have limited the treatment of malaria [3,4]. Artemisinin and its derivatives, broadly known as Artemisinins (ARTs), a new class of compounds derived from the Chinese herbal plant Qinghao and discovered by Youyou Tu in the 1970s, have been the most potent and useful frontline drugs for the treatment of drug-resistant malaria available to date [5,6,7]. Chemically, ARTs are attributed to the sesquiterpene lactone class, having an endoperoxide bridge which is pharmacologically significant for anti-malarial activity [8]. Among the ART analogs, artesunate (AS) demonstrated the most versatile semisynthetic derivative due to its broader therapeutic potential, rapid action, and better water solubility [9,10,11]. Apart from its wide therapeutic use, AS exhibits a short plasma half-life and low bioavailability, which undermine the effectiveness of optimal therapeutic efficacy [12]. Moreover, AS monotherapy becomes less effective due to the emergence of parasite resistance [13]. Therefore, the WHO introduced artemisinin-based combination therapies (ACTs), in which fast-acting artesunate is administered together with slow-acting antimalarial drugs of different classes to combat resistance and improve the effectiveness of the treatment [10,14,15,16,17]. Nevertheless, recent studies demonstrated a high rate of ACT failure, which might be due to the substandard quality of drugs, inappropriate use of monotherapies, and prematurely stopping of ACT therapy. This leads often to parasite recrudescence in the Greater Mekong Subregion (GMS) [18].

Over the years, many studies have been conducted to find a way to increase the potency of antimalarial drugs [19]. One of the most promising concepts is the synthesis of new efficient artemisinin therapeutics through the dimerization technique [20]. This technique has gained great interest due to the increase in antimalarial potency and provides new strategies for drug discovery and development [21]. Dimerization refers to the process of combining two identical molecules, which may lead to the development of novel compounds with improved pharmacological properties compared to parent compounds [22]. Many ART dimers have been reported by several groups. Posner et al. introduced a series of artemisinin-derived dimers that possess potential antimalarial activity both in vitro and in vivo [23,24,25]. It was also reported that ART trioxane dimers showed complete parasitic clearance with a prolonged survival time of the malaria-infected mice model [26]. Reiter et al. prepared a series of ART dimers and trimers and assessed their efficiency against malarial 3d7 strains. The dimeric derivatives showed superior antimalarial activity compared to the trimer and parent compound [27]. Several ART triazine hybrids and hybrid dimers were synthesized by Cloete et al. which exhibit effectiveness against chloroquine-sensitive (CQS) and chloroquine-resistant (CQR) Dd2 strains of the malaria parasite [28].

Furthermore, other studies have been directed toward the field of nanotechnology to resolve the challenges of the conventional antimalarial dosage form such as poor water solubility, short half-life, low bioavailability, multiple drug dose, and high drug-related toxicity. Nanocarriers can play an essential role in the case management of malaria by altering the pharmacokinetics and pharmacodynamics of drugs [29]. In this respect, several ART-based drug delivery systems have been investigated, such as implants [30], liposomes, polymeric nanoparticles, lipid-based nanoparticles, metal-based nanoparticles, hybrid drugs, and nanoemulsion (NE) [31]. Among them, NE has been explored extensively as an appropriate carrier to improve the pharmacokinetics and to release the drug in a controlled manner [32]. The NE can protect the active ingredient from oxidative and hydrolytic degradation [33].

In the current work, two well-known strategies were combined into one system. Firstly, dimerization of the drug to develop a new dimeric entity, and, secondly, utilization of this new conjugate for the formulation of nanoemulsion preconcentrate. In short, we synthesized a dimeric artesunate glycerol monocaprylate conjugate (D-AS-GC) and designed a nanocarrier-based lipid NE. Initially, AS was conjugated with a linker molecule namely glycerol monocaprylate (GC) via simple esterification reaction to obtain a D-AS-GC conjugate. AS, GC, and D-AS-GC conjugate were characterized by ^1^H NMR, attached proton test (APT) ^13^C NMR, 2D NMR such as homonuclear correlation spectroscopy (COSY), heteronuclear single quantum coherence (HSQC), and heteronuclear multiple bond correlation (HMBC) spectroscopy. The molar mass of the conjugate was confirmed by electrospray ionization time-of-flight mass spectroscopy (ESI-TOF MS). Afterwards, different formulations of D-AS-GC loaded nanoemulsion preconcentrate were developed. The particle size of all formulations was measured by dynamic light scattering (DLS). Finally, the particle size of optimized nanoformulation was further characterized by diffusion-ordered spectroscopy (DOSY). Atomic force microscopy (AFM) was used to investigate the final morphology of drying emulsion droplets on different substrates.

## 2. Results and Discussion

### 2.1. Synthesis and Characterization of the D-AS-GC Conjugate

**Scheme 1:**
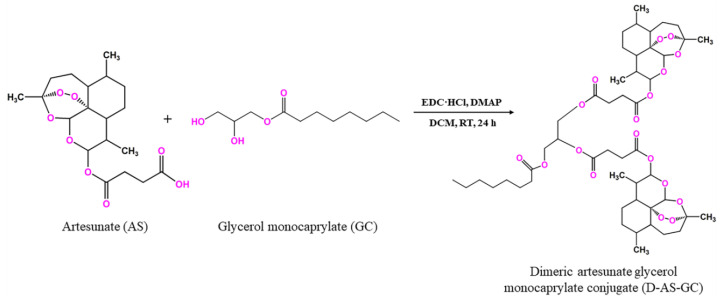
Synthetic pathway for dimeric artesunate glycerol monocaprylate conjugate (D-AS-GC).

Generally, the synthesis of ART dimers can increase the selectivity for target binding, reduce toxicity, and overcome drug resistance, leading to the design of more effective and safer antimalarial agents [19]. Accordingly, dimeric artesunate glycerol monocaprylate conjugate (D-AS-GC) was achieved by the formation of the ester bond between artesunate (AS) and glycerol monocaprylate (GC) via a facile esterification reaction in the presence of EDC·HCl and DMAP as a coupling agent and a catalyst, respectively, as shown in Scheme 1. Here, the GC linker was chosen to conjugate artesunate dimer, which, in turn, results in an increase in compatibility with the formulation of lipid-based nanoemulsion preconcentrate [34].

**Figure 1:**
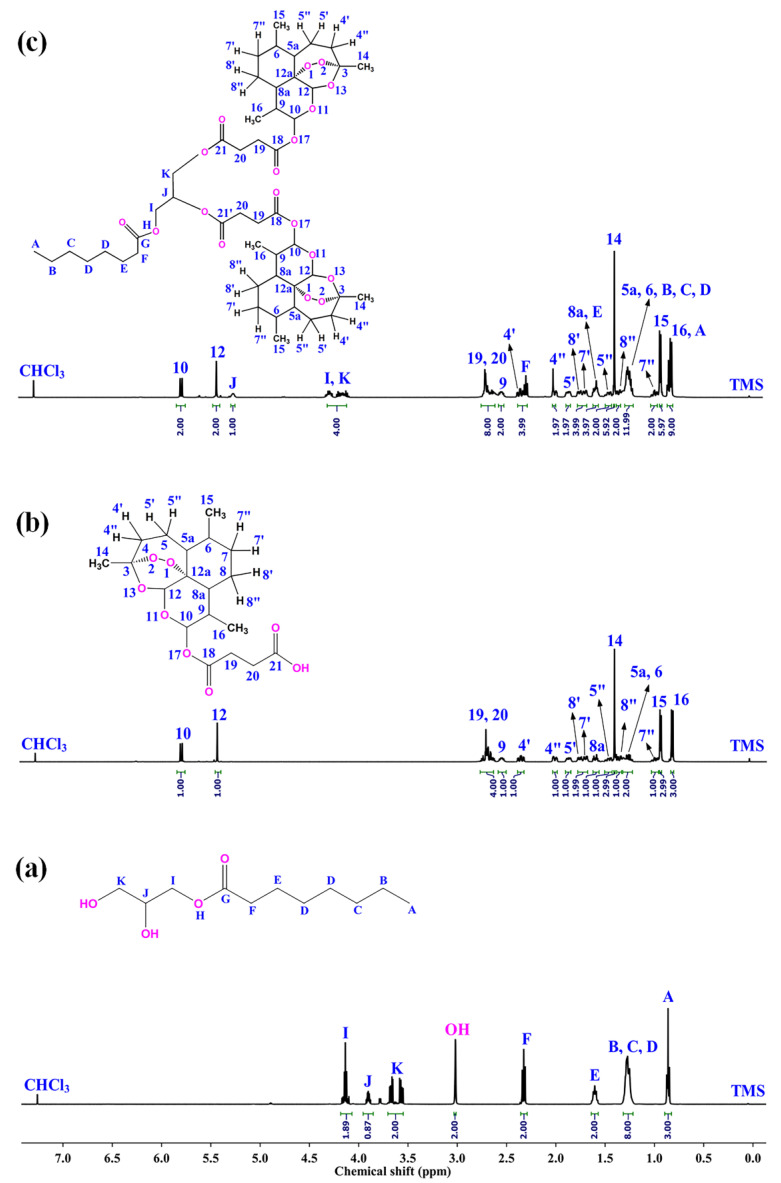
The ^1^H NMR spectra of (**a**) GC, (**b**) AS, and (**c**) D-AS-GC were recorded at 27 °C, 500 MHz, using CDCl_3_ as solvent.

**Figure 2:**
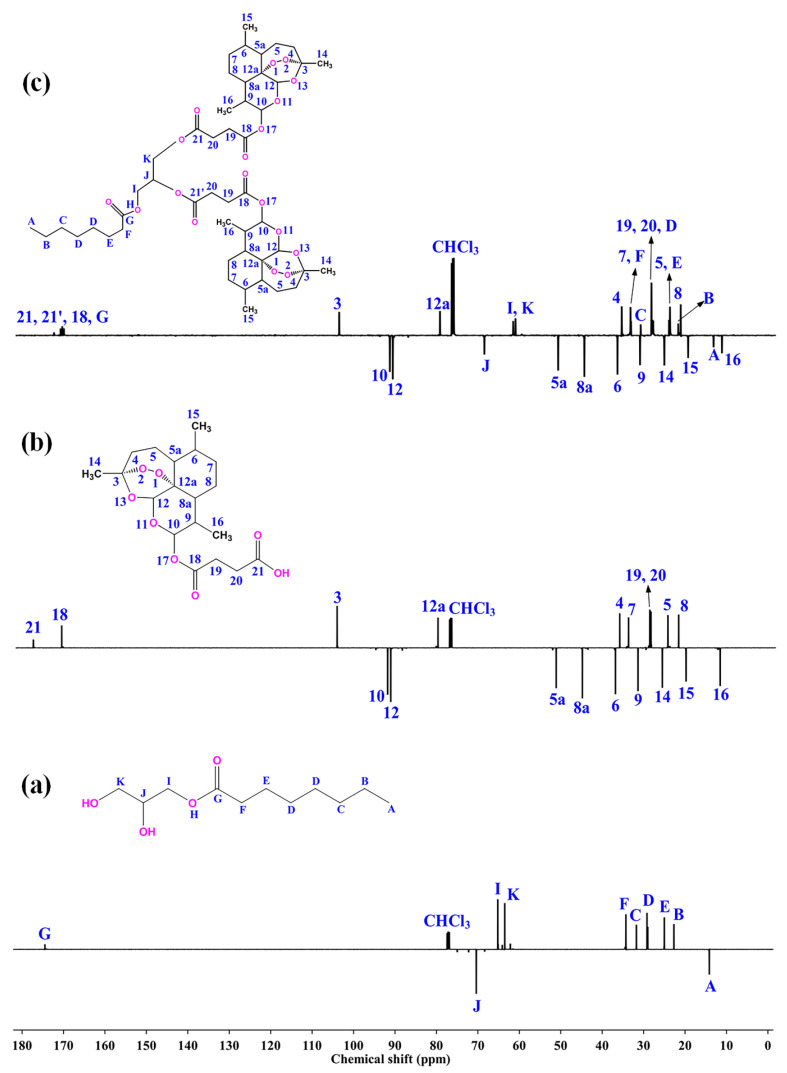
APT ^13^C NMR spectra of (**a**) GC, (**b**) AS, and (**c**) D-AS-GC were recorded at 27 °C, 125 MHz, using CDCl_3_ as solvent.

The conjugation product D-AS-GC was characterized by ^1^H NMR, APT ^13^C NMR, and 2D HSQC NMR spectroscopy. The ^1^H NMR spectra of GC, AS, and D-AS-GC are shown in Figure 1a–c, respectively. The comparison between ^1^H NMR spectra a and c in Figure 1 shows a significant downfield shift of the resonance signal (labeled as J) from 3.92–3.88 ppm to 5.28–5.23 ppm, which is attributed to the methine proton of GC, as well as a downfield shift of the proton resonances (labeled as K) from 3.69–3.55 ppm to a higher overlap signal at 4.32–4.09 ppm assigned as methylene protons of GC. Additionally, a complete disappearance of the proton signal of the hydroxyl groups (–OH) of GC is observed, which indicates the successful conjugation of AS with GC. Besides ^1^H NMR, APT ^13^C NMR spectra of GC, AS, and D-AS-GC are given in Figure 2a–c, respectively. Comparing APT ^13^C NMR spectra a and c in Figure 2 reveals upfield shifts of carbon signals from 70.38 ppm to 69.37 ppm (peak J) and 63.52 ppm to 62.41–61.86 ppm (peak K), which belong to the methine and methylene carbons of GC. Further, no carbonyl signal (C=O) of the carboxylic acid group (peak 21) of AS is observed in Figure 2c in comparison with Figure 2b, thereby suggesting the efficient formation of D-AS-GC conjugate. Additionally, the chemical structure of AS, GC, and D-AS-GC conjugate was further confirmed by 2D NMR spectroscopy (given in the Supplementary Materials in Figures S1–S7 and Tables S1–S7).

**Figure 3:**
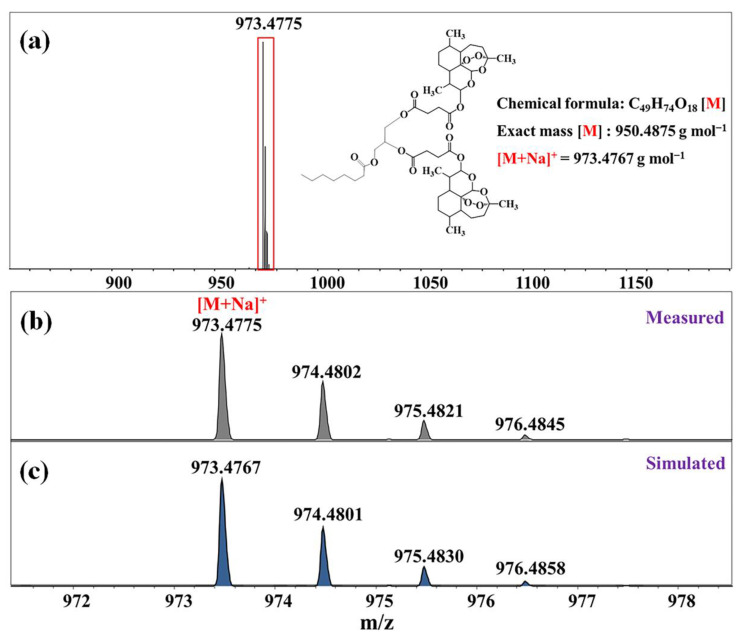
ESI-TOF MS spectra of D-AS-GC. (**a**) Full spectrum, (**b**) region of the main peak, and (**c**) simulated isotope signals of the marked section. Inset shows the structure of D-AS-GC with the respective molar mass.

The molar mass of the D-AS-GC conjugate was determined by electrospray ionization time-of-flight mass spectroscopy (ESI-TOF MS), which is a soft ionization technique used for the precise determination of the molecular mass of organic molecules [35]. The ESI-TOF MS spectrum in Figure 3a shows a major molecular ion peak at *m*/*z* 973.4775 (M+Na)^+^, which matches with the simulated isotopic patterns for the theoretically calculated value *m*/*z* 973.4767. The neighboring mass peaks (Figure 3b) follow the natural isotope distribution (with binomial distributed 1.1% ^13^C) as indicated by the simulated pattern (Figure 3c).

### 2.2. Preparation and Characterization of Nanoemulsion

Nanoemulsion preconcentrate is a combination of drugs and lipophilic excipients as an oil phase with water-soluble surfactants and cosurfactants. It forms clear, stable small droplets of o/w nanoemulsion upon dilution by an aqueous phase [36]. All the excipients selected here are based on a saturated chemical structure because unsaturated compounds are very prone to undergo oxidative degradation [37]. Medium-chain triglyceride (MCT) was chosen as the oil phase due to its superior physical, pharmaceutical, and clinical attributes [38]. Kolliphor HS 15 and propylene glycol were selected as surfactant and cosurfactant, respectively. Kolliphor HS 15 is mainly designed for parenteral applications owing to its good safety margin [39]. The dimeric artesunate conjugates (D-AS-GC) were used to prepare in total six different formulations (see Table 1) namely, F1 (1:1:2), F2 (1:1:3), F3 (1:1:4), F4 (1:2:4), F5 (1:2:6), and F6 (1:2:8) by varying the mass ratios (w/w) of D-AS-GC to MCT to Kolliphor HS 15/propylene glycol, respectively, to optimize a transparent nanoemulsion upon dispersing into an aqueous phase. The Kolliphor HS 15/propylene glycol mass ratio of 2:1 (w/w) was kept unchanged for all formulations.

**Figure 4:**
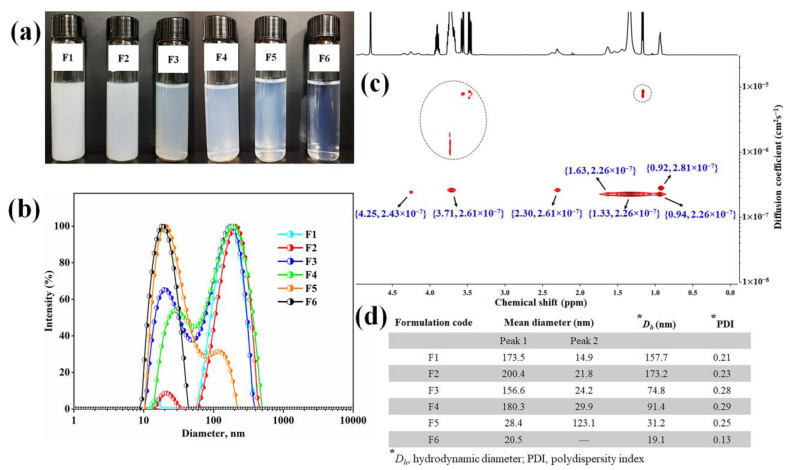
Characterization of D-AS-GC nanoemulsions. (**a**) The visual appearance and (**b**) DLS measurements of nanoemulsion formulations F1, F2, F3, F4, F5, and F6. (**c**) DOSY NMR of formulation F6 (Inset shows the ^1^H chemical shift and diffusion coefficient). (**d**) Summary of the mean diameter, hydrodynamic diameter, and polydispersity index of different formulations (F1–F6) analyzed by DLS.

The visual observation of turbidity is a simple method to identify clear nanoemulsion formulation. In Figure 4a, it is observable that the optical appearance of the formulations changed from white (F1) to a transparent or slightly opalescent (F6) appearance. The shift of turbidity to clear transparency can be described in terms of thermodynamics and kinetic phenomena of nanoemulsions. Generally, nanoemulsion formation is a non-equilibrium and non-spontaneous process because the total Gibbs free energy required for the formation of nanoemulsion is positive [40]. A surfactant/cosurfactant assists the kinetic stability of nanoemulsion by reducing the interfacial tension between oil and water, leading to a decrease in Gibbs free energy [41]. Hence, an increase in the surfactant/cosurfactant amount leads to a decrease in droplet size by increasing the surface to volume ratio, which, in turn, reduces the scattering of the incident light and gives rise to visually clear nanoemulsions [42,43]. The opalescence is another indication of Rayleigh scattering that confirms the stable nanoemulsion formation. It occurs when the droplet size of the nanoemulsion is less than the wavelength of incident light. Since violet-blue light with a wavelength of 400 nm scatters more than red light, the nanoemulsion droplet size of less than 100 nm shows a blue tint in the formulation [44]. Overall, the change in turbidity is directly related to the droplet size of the formulations.

To make a relationship between turbidity with the particle size and polydispersity index, the DLS technique is used for all formulations (Figure 4b,d). In the case of formulation F1 to F3, the D-AS-GC to MCT ratio is constant at 1:1, but MCT to Kolliphor HS 15/propylene glycol ratios increase gradually from 1:2 (F1) to 1:3 (F2) and 1:4 (F4) (see Table 1), which produce a white to opaque appearance of the formulations. In addition, the hydrodynamic diameter (*D_h_*) of the particles decreases from 157.8 nm (F1) to 74.8 nm (F3) while the polydispersity index (PDI) increases from 0.21 (F1) to 0.28 (F3) (Figure 4d). In formulations F4 to F6 (see Table 1), the D-AS-GC to MCT ratio is 1:2, and MCT to Kolliphor HS 15/propylene glycol ratios increase accordingly from 2:4 (F4) to 2:6 (F5) and 2:8 (F6). Formulations F4 to F6 appear translucent to clear. This indicates that the oil phase is sufficient to completely dissolve the drugs (D-AS-GC). Consequently, the *D_h_* of formulation F4 to F6 (Figure 4d) decreases from 91.4 nm to 19.1 nm, and the PDI decreases from 0.29 to 0.13. The decrease in hydrodynamic diameter is due to the increase in surface to volume ratio with an increase in the surfactant/cosurfactant ratio in the formulations. It is known that Kolliphor HS 15 forms micelles with a diameter of about 12 nm [45]. The diameter of F6 is only slightly higher. This formulation could also be regarded as swollen micelles. With increasing loading of hydrophobic liquids, micelles transform into swollen micelles and finally into emulsions. The above observations demonstrate that oil and surfactant/cosurfactant ratios have a great influence on the solubility of D-AS-GC and the formation of a clear nanoemulsion. Though the particle size decreases with increasing surfactant/cosurfactant ratios to D-AS-GC and oil, the formulations (F1, F2, F3, F4, and F5) exhibit bimodal distributions in DLS measurements, which indicates the existence of two important populations of droplets (Figure 4b,d). The Table in Figure 4d represents the mean diameter of the droplets (F1–F6) and the peak numbering is based on the respective total area under each peak (peak 1 is the largest and peak 2 is the smallest area under the peaks). Only formulation F6 shows a monomodal particle size distribution with a very narrow PDI of 0.13 (Figure 4b,d). Hence, formulation F6 (1:2:8) was selected as the best combination for nanoemulsion formulation.

Two-dimensional diffusion-ordered nuclear magnetic resonance spectroscopy (DOSY NMR) was further used to determine the hydrodynamic diameter of droplets in the F6 formulation. DOSY NMR is a sensitive and noninvasive tool to determine the hydrodynamic diameter of the macromolecular complexes in the dispersion medium [46]. It provides a 2D NMR plot that represents a ^1^H chemical shift along the x-axis and the diffusion coefficient along the y-axis. The cross peak in DOSY NMR indicates the diffusion coefficient of the molecule corresponding to the ^1^H NMR shifts [47]. The hydrodynamic diameter is calculated from the Stokes–Einstein equation, $D_{h}=k_{B}T/3\pi \eta D$, where $D_{h}$ is the hydrodynamic diameter (m), $k_{B}$ is the Boltzmann constant (1.38 × 10^−23^ kgm^2^s^−2^K^−1^), *T* is the absolute temperature (298.15 K), *η* is the viscosity of the continuous phase D_2_O (0.00125 kgm^−1^s^−1^ at 298.15 K), and *D* is the diffusion coefficient (cm^2^s^−1^). The DOSY NMR spectrum of the F6 formulation is shown in Figure 4c. From the ^1^H NMR spectra of D-AS-GC (Figure 1c), MCT, Kolliphor HS 15, and propylene glycol (shown in Figure S8), it is confirmed that most of strong DOSY NMR signals correspond to MCT, Kolliphor HS 15, and propylene glycol. All the resonances allow us to calculate the diffusion coefficient of around 2.26–2.81 × 10^−7^ cm^2^s^−1^. Consequently, the hydrodynamic diameter was calculated from the Stokes–Einstein equation and ranged from around 13 nm to 16 nm. Small cross peaks in the DOSY NMR spectrum (dashed circle in Figure 4c) in the range of 1 × 10^−5^ to 1 × 10^−6^ cm^2^s^−1^ represent the diffusion coefficient of propylene glycol or tiny aggregates present in the formulation. The measured oil droplet size (*D_h_*) of 13–16 nm is close to the value of 19.1 nm obtained by DLS.

**Figure 5:**
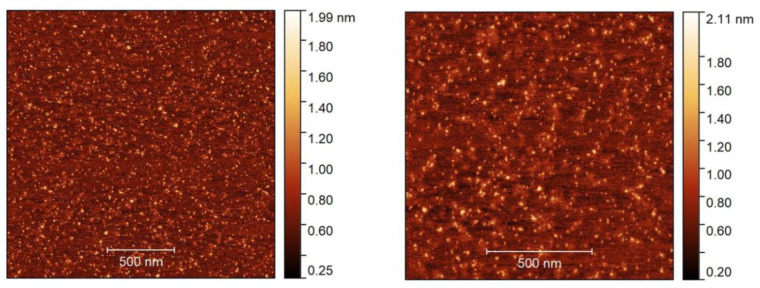
AFM height images in two different sizes, 2 × 2 µm (**left**) and 1.3 × 1.3 µm (**right**), with a sample (F6) concentration of 0.01 mg/mL dried on the mica substrate.

To examine the morphology of nanoemulsion (formulation F6), we dispersed the prepared sample of different concentrations on two different substrates, i.e., mica or silicon wafer, dried and checked by AFM. Figure 5 shows the morphology of F6 prepared with a concentration of 0.01 mg/mL and dried on mica. The dark reddish color represents the mica surface, whereas the bright color depicts the morphology of the nanoemulsion droplets.

**Figure 6:**
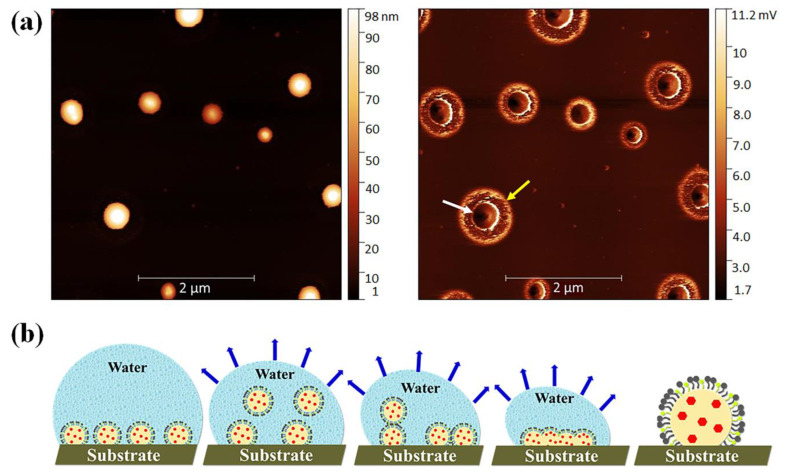
(**a**) AFM height (**left**) and adhesion (**right**) image with a size of 5 × 5 µm and with a sample (F6) concentration of 0.05 mg/mL dried on the silicon substrate, and (**b**) schematic presentation of the coalescence process of the oil droplets during drying on the substrate.

AFM reveals the presence of small droplets in the dried nanoemulsion with a dimension ranging from ~10 to 50 nm with an average height of ~2 nm. The actual morphology of the nanoemulsion is hard to resolve due to the soft or sticky nature of the droplets which generate artifacts and become deformed during imaging [48]. Hence, a typical core shell-like structure of o/w droplets was not observed in the height images or even in other imaging modes (e.g., adhesion or modulus). The droplet size obtained from DLS and DOSY NMR spectroscopy, however, was in quite good agreement with the AFM measurement. In the next step, we spread a slightly higher concentration (0.05 mg/mL) of the formulation F6 on a silicon wafer and dried it. Figure 6a visualizes the different images (left: height, right: adhesion) of the droplets. The AFM image of the F6 formulation shows a distinct spherical core (denoted by a white arrow) and shell (marked by a yellow arrow) morphology of the droplets. The dark color is observed in the inner cavity, which is composed of drug molecules (D-AS-GC), and a slightly bright color is observed around the core, which is the shell made of MCT (oil) and Kolliphor HS 15/propylene glycol (surfactant/cosurfactant). However, the dimensions of the droplets are in the range of ~200 nm to 2 µm with an average height of ~100 nm, which is much larger than the droplet size found on the mica surface. This is because the silicon wafer is a less polar substrate, and spreading a slightly higher concentration of the sample on the substrate, along with solvent evaporation, leads to the formation of large droplets due to the evaporation-induced coalescence of oil droplets [49]. A schematic diagram of the coalescence process of the oil droplets during drying on the substrate is shown in Figure 6b. The above findings provide evidence of the formation of nanoemulsion with a well-defined inner core of D-AS-GC surrounded by a spherical homogenous shell composed of oil and surfactant/cosurfactant.

The dimerization technique used to synthesize D-AS-GC conjugate may potentially improve the potency of the drug, increase bioavailability with a minimum dose of drug, reduce side effects, and overcome the drug resistance. Afterwards, the synthesized conjugate was used to develop D-AS-GC loaded nanoemulsion preconcentrate. Comparing with the previous reports, the advantages of nanoemulsions over the conventional drug delivery systems is that small droplets increase the kinetical stability of nanoemulsions. Moreover, a small droplet size of the nanoemulsions provides a large surface area, which enhances the penetration of active ingredients and increases the bioavailability of drugs. The utilization of nanoemulsions enables a reduction in the required drug dosage by enhancing bioavailability, extending retention time within the body, and minimizing drug loss. Nanoemulsion could also increase chemical stability of drugs by protecting them from oxidation and hydrolysis.

## 3. Materials and Methods

### 3.1. Materials

Artesunate (97%) was purchased from abcr GmbH (Karlsruhe, Germany). Glycerol monocaprylate (type II, IMWITOR^®^ 308) and medium-chain triglycerides (MIGLYOL^®^ 812 N) were purchased from IOI Oleo GmbH (Hamburg, Germany). 4-(dimethylamino)pyridine (DMAP), silica gel (SiO_2_, 0.03–0.2 mm), 1-ethyl-3-(3-dimethylaminopropyl)carbodiimide hydrochloride (EDC·HCl), deuterium oxide (D_2_O, 99.9%), deuterated chloroform (CDCl_3_, 99.8%), HPLC grade *n*-hexane, and ethyl acetate were purchased from Carl Roth (Karlsruhe, Germany). Dichloromethane (DCM, anhydrous, 99.9%) was obtained from Acros Organics (Schwerte, Germany). Kolliphor^®^ HS 15 (Polyoxyl (15) hydroxystearate) was obtained from BASF (Ludwigshafen, Germany). Propylene glycol (≥99.5%, GC grade) was purchased from Fluka Chemie AG (Buchs, Switzerland).

### 3.2. Methods

#### 3.2.1. Nuclear Magnetic Resonance (NMR) Spectroscopy

The NMR spectra were recorded on a VNMRS spectrometer (Agilent Technologies, Santa Clara, CA, USA) at 500 MHz for ^1^H NMR and 125 MHz for the attached proton test (APT) ^13^C NMR. Two-dimensional NMR measurements, namely, homonuclear correlation spectroscopy (COSY), heteronuclear single quantum coherence (HSQC) spectroscopy, and heteronuclear multiple bond correlation (HMBC) spectroscopy, were performed to obtain detailed structural elucidation. A total of 20 mg of samples were dissolved in 0.6 mL of deuterated solvents (CDCl_3_ and D_2_O). The solvent residual signal for CHCl_3_ was set at 7.26 ppm for ^1^H, 77.0 ppm for the APT ^13^C NMR spectra, and 4.79 ppm for HDO in the ^1^H NMR spectrum. All measurements were carried out at 27 °C, using tetramethylsilane (TMS) as an internal standard. The NMR spectral data were interpreted using MestRec (v.4.9.9.6) software (Mestrelab Research, Santiago de Compostela, Spain).

#### 3.2.2. Electrospray Ionization Time-of-Flight Mass Spectroscopy (ESI-TOF MS)

A Focus Micro TOF spectrometer from Bruker Daltonics (Billerica, MA, USA) was used to conduct ESI-TOF MS measurements. A total of 1 mg of the sample was dissolved in HPLC grade THF at a final concentration of 10 µL/mL. Then, 10 µL of sodium iodide (NaI) was added to the sample solution. The samples were injected with a flow rate of 180 µL/h. An acceleration voltage of 4.5 kV was used to record the spectra in positive mode. The measured data were interpreted using Data Analysis 4.2 software from Bruker Daltonics.

#### 3.2.3. Thin-Layer Chromatography (TLC)

Detection of the conjugate formation was performed by thin-layer chromatography (TLC) using the precoated silica gel aluminum sheet (Merck silica gel 60) as the stationary phase. A total of 5–10 µL of the sample was spotted on the TLC plate and immersed in the mobile phase containing a mixture of solvents (ethyl acetate:*n*-hexane 4:10) in a way that the spot remained above the level of the mobile phase. Afterwards, the TLC plate was removed and dried. The spot of the new conjugate was visualized by iodine vapor.

#### 3.2.4. Synthesis of Dimeric Artesunate Glycerol Monocaprylate Conjugate (D-AS-GC)

Dimeric artesunate glycerol monocaprylate conjugate (D-AS-GC) was synthesized via a simple one-step Steglich esterification reaction [50,51]. Briefly, 0.5 g (2.29 mmol) of glycerol monocaprylate (GC) was dissolved in 40 mL of ice-cooled anhydrous DCM in a 100 mL 3-neck round-bottom flask equipped with a magnetic stirrer. Afterwards, 2.64 g (6.86 mmol, 3 eq. of –OH groups) of artesunate (AS), 1.3 g of EDC∙HCl (6.87 mmol, 3 eq.), and 0.084 g of DMAP (0.687 mmol, 0.3 eq.) were added to the solution. The reaction was carried out at 0 °C for 30 min followed by 24 h at room temperature under constant stirring. The whole reaction was performed under inert conditions. The synthesized product was purified by column chromatography using a mixture of ethyl acetate and n-hexane (4:10) as mobile phase. The desired product was separated and confirmed by TLC. The pure fractions were collected and dried under reduced pressure by rotary evaporator. The resultant product was kept under high vacuum to provide D-AS-GC conjugate as white solid. ESI-TOF MS (*m*/*z*): calculated for C_49_H_74_O_18_, 950.4875 g mol^−1^ [M]; found, 973.4775 g mol^−1^ [M+Na]^+^. The ^1^H NMR (D-AS-GC, 500 MHz, CDCl_3_): *δ* (ppm) 5.78–5.76 (2H, H-10), 5.43–5.40 (2H, H-12), 5.28–5.23 (1H, H-J), 4.32–4.09 (4H, H-I, K), 2.73–2.62 (8H, H-19, 20), 2.57–2.50 (2H, H-9), 2.39–2.29 (4H, H-4′, F), 2.04–1.99 (2H, H-4″), 1.90–1.86 (2H, H-5′), 1.78–1.69 (4H, H-8′, 7′), 1.62–1.58 (4H, H-8a, E), 1.50–1.45 (2H, H-5″), 1.42–1.39 (6H, H-14), 1.39–1.34 (2H, H-8″), 1.31–1.23 (12H, H-5a, 6, B, C, D), 1.04–0.97 (2H, H-7″), 0.96–0.93 (6H, H-15) and 0.88–0.83 (9H, H-16, A). APT ^13^C NMR (D-AS-GC, 125 MHz, CDCl_3_): *δ* (ppm) 173.24–171.66 (C-21, 21′, 18, G), 104.42 (C-3), 92.19 (C-10), 91.48 (C-12), 80.09 (C-12a), 69.37 (C-J), 62.41–61.86 (C-I, K), 51.57 (C-5a), 45.24 (C-8a), 37.25 (C-6), 36.22 (C-4), 34.10–33.98 (C-7, F), 31.80 (C-9), 31.63 (C-C), 29.04–28.62 (C-19, 20, D), 25.93 (C-14), 24.84–24.58 (C-5, E), 22.51(C-B), 21.98 (C-8), 20.19 (C-15), 14.05 (C-A) and 12.03 (C-16). The ^1^H NMR (AS, 500 MHz, CDCl_3_): *δ* (ppm) 5.78–5.76 (1H, H-10), 5.43–5.40 (1H, H-12), 2.73–2.62 (4H, H-19, 20), 2.57–2.50 (1H, H-9), 2.39–2.33 (1H, H-4′), 2.04–1.99 (1H, H-4″), 1.90–1.86 (1H, H-5′), 1.78–1.69 (2H, H-8′, 7′), 1.62–1.58 (1H, H-8a), 1.50–1.45 (1H, H-5″), 1.42–1.39 (3H, H-14), 1.39–1.34 (1H, H-8”), 1.31–1.23 (2H, H-5a, 6), 1.04–0.97 (1H, H-7″), 0.96–0.93 (3H, H-15) and 0.88–0.83 (3H, H-16). APT ^13^C NMR (AS, 125 MHz, CDCl_3_): *δ* (ppm) 177.94 (C-21), 171.10 (C-18), 104.42 (C-3), 92.39 (C-10), 91.63 (C-12), 80.22 (C-12a), 51.68 (C-5a), 45.36 (C-8a), 37.38 (C-6), 36.33 (C-4), 34.21 (C-7), 31.92 (C-9), 29.06–28.79 (C-19, 20), 26.04 (C-14), 24.69 (C-5), 22.10 (C-8), 20.31 (C-15) and 12.08 (C-16). The ^1^H NMR (GC, 500 MHz, CDCl_3_): *δ* (ppm) 4.17–4.10 (2H, H-I), 3.92–3.88 (1H, H-J), 3.69–3.55 (2H, H-K), 3.02 (–OH), 2.35–2.31 (2H, H-F), 1.64–1.58 (2H, H-E), 1.32–1.21 (8H, H-B, C, D) and 0.88–0.85 (3H, H-A). APT ^13^C NMR (GC, 125 MHz, CDCl_3_): *δ* (ppm) 174.49 (C-G), 70.38 (C-J), 65.20 (C-I), 63.52 (C-K), 34.27 (C-F), 31.74 (C-C), 29.19 (C-D), 25.00 (C-E), 22.69 (C-B) and 14.14 (C-A).

#### 3.2.5. Formulation of Nanoemulsion Preconcentrate and Optimization

Nanoemulsion preconcentrate is an anhydrous system composed of drug, oil, surfactant, and cosurfactant. It spontaneously forms oil in water (o/w) nanoemulsion upon dispersing in an aqueous phase [52]. Accordingly, in this study, the nanoemulsion preconcentrates were prepared which were composed of D-AS-GC, medium-chain triglycerides (MCT), Kolliphor HS 15/propylene glycol as drug, oil, and surfactant/cosurfactant. The preconcentrates were prepared according to Yao-Xing Dou et al. with a slight modification [53]. The composition of different nanoemulsion preconcentrates is presented in Table 1, keeping the surfactant/cosurfactant ratio of 2:1 (w/w) constant for all formulations. Predetermined ratios of D-AS-GC, MCT and Kolliphor HS 15/propylene glycol were taken in a small vial and mixed under gentle magnetic stirring at 40 °C until a clear solution was obtained. Afterwards, the preconcentrate was cooled down to room temperature and kept in the air-tight vial and dispersed in double distilled water for nanoemulsion formulation. All the nanoemulsion preconcentrates were further examined visually and by dynamic light scattering (DLS) after being dispersed into the double distilled water to optimize a homogeneous and clear nanoemulsion formulation.

#### 3.2.6. Visual Evaluation of the Formulations

The prepared nanoemulsion preconcentrates were diluted at 1:30 with double distilled water and gently mixed by a magnetic stirrer. The resulting mixtures were equilibrated for 30 min at room temperature and the appearance of the formulations was visually examined.

#### 3.2.7. Determination of Droplet Size by Dynamic Light Scattering (DLS)

Hydrodynamic diameter (*D_h_*) and intensity-weighted size distribution of the droplets in the formulations were analyzed by DLS using Litesizer 500 device (Anton Paar GmbH, Graz, Austria). The formulations were taken from the previous experiment and poured carefully into a quartz cell (Hellma Analytics, Müllheim, Germany) to avoid air bubbles. The measurements were performed at a light wavelength of 658 nm, a detection angle of 175° (backscattering), and the temperature was set to 25 °C. *D_h_* and the broadness of the intensity size distribution, called the polydispersity index (PDI), were derived from the intensity curve fitting by applying the autocorrelation function using Kalliope Software (Anton Paar GmbH).

#### 3.2.8. Droplet Size Measurement Using Diffusion-Ordered NMR Spectroscopy (DOSY NMR)

Based on the results of the visual evaluation and DLS measurements, the hydrodynamic diameter of the optimized sample was further analyzed by DOSY NMR spectroscopy. The DOSY NMR sample was prepared using D_2_O in place of H_2_O as the dispersing phase of the desired nanoemulsion preconcentrate. The DOSY NMR spectrum was recorded in an Agilent VNMR DD2 (500 MHz, version OpenVnmr 2.1) spectrometer. The measurement was obtained using the Agilent pulse program DgcsteSL_cc. The diffusion delay and relaxation delay were set at 300 ms and 2 s, respectively, with a diffusion gradient length of 3 ms and a total number of 16 scans. The experiment was performed at 27 °C and the solvent residual signal of HDO was set at 4.79 ppm in the ^1^H NMR spectrum.

#### 3.2.9. Morphological Analysis by Atomic Force Microscopy (AFM)

A Bruker MultiMode 8 AFM instrument was used to check the droplet dimensions in the nanoemulsions. The samples were prepared in two different concentrations, 0.01 and 0.05 mg/mL, and were applied at a volume of 10 to 20 µL using a Hamilton syringe on two different substrates, e.g., a freshly cleaved mica surface and a CO_2_ snow jet cleaned silicon plate of 10 mm^2^. After solvent evaporation, the substrates were carefully examined by optical microscopy, and the area of interest was scanned by AFM. Images were acquired in PeakForce Quantitative Nanomechanics (PF-QNM) tapping mode using a SCANSYST-AIR cantilever with a spring constant of 0.4 N m^−1^ and a resonance frequency of 70 kHz. The images were recorded with a cantilever oscillation of 2 kHz and a scan speed of 0.5 Hz. The PF-QNM modes provide six different images simultaneously, such as height, peak force error, modulus, adhesion, deformation, and dissipation. Only height and adhesion images are presented here. The height image provides information about the height and lateral dimensions, whereas the adhesion image provides the adhesion properties of the sample relative to the cantilever, meaning that a strong contrast can be seen when the cantilever and sample have higher adhesion. This helps to identify various morphologies that cannot be resolved well in the height image. However, as the cantilever was not calibrated, the adhesion force in the image appears as volt rather than newton. Finally, the captured images were then processed by Gwyddion software (open source software, Czech Metrology Institute, Brno, Czech Republic).

## 4. Conclusions

In this paper, the dimerization technique and the nanoemulsion formulation were combined to comprise the properties of both approaches into a drug delivery system in order to increase the potency and efficacy of artesunate-based antimalarial treatment. Recently, an amphiphilic dimeric artesunate glycerophosphorylcholine (Di-ART-GPC) liposome was developed, which showed longer retention time and enhanced bioavailability in vivo compared to the parent drug [54,55]. Accordingly, we described the covalent conjugation of artesunate (AS) with a linker molecule glycerol monocaprylate (GC) to synthesize dimeric artesunate glycerol monocaprylate conjugate (D-AS-GC), which was characterized by ESI-TOF MS and 1D and 2D NMR spectroscopy. Later, different D-AS-GC loaded nanoemulsion preconcentrates were developed to optimize a transparent, stable nanoemulsion formulation upon dilution with an aqueous phase (double distilled water). Formulation F6 showed a clear nanoemulsion with a monomodal particle size distribution and a very narrow PDI of 0.13. The morphology of F6 was investigated by AFM using two different substrates to understand nanoemulsion characteristics and to visualize the internal structure of the nanoemulsion droplets. AFM results revealed the formation of nanodroplets close to DLS and DOSY NMR values with no observed defined structure on the mica surface. However, upon deposition of the formulation and drying on the silicon surface, an inner cavity surrounded by a spherical outer shell was observed, which confirmed the formation of a core shell-like structure composed of an inner core of drug molecules (D-AS-GC) with a distinct outer layer of MCT (oil) and Kolliphor HS 15/propylene glycol (surfactant/cosurfactant). In conclusion, we successfully synthesized dimeric artesunate glycerol monocaprylate conjugate (D-AS-GC) and developed a nanocarrier system for D-AS-GC delivery, thus creating a promising drug candidate for antimalarial treatment in combination with other drugs or as monotherapy. This strategy can further help to achieve an extended drug circulation time, increase bioavailability with a minimum dose of drugs, and control drug release over a long period of time. However, further research is necessary to verify the structure–activity relationship (SAR), pharmacokinetic and pharmacodynamic properties, and overall pharmacological and toxicological profile of the formulation.

## Figures and Tables

**Table 1:** Formulation composition ratios (*w*/*w*) of the different nanoemulsion preconcentrate.

Formulation Code	D-AS-GC (Drug)	MCT (Oil)	Kolliphor HS 15/Propylene Glycol (2:1) (Surfactant/Cosurfactant)
F1	1	1	2
F2	1	1	3
F3	1	1	4
F4	1	2	4
F5	1	2	6
F6	1	2	8

## Data Availability

The data presented in this article are available on request from the corresponding author.

## Supplementary Materials

The following supporting information can be downloaded at: https://www.mdpi.com/article/10.3390/molecules28135208/s1, Figure S1: HSQC NMR spectrum of AS was recorded at 27 °C using CDCl_3_ as solvent. The inset shows the HSQC NMR spectrum of AS in the range of 0–8 ppm and 90–180 ppm for ^1^H and APT ^13^C NMR chemical shifts, respectively. No additional correlation signals are observed in this region; Figure S2: COSY NMR spectrum of AS was recorded at 27 °C using CDCl_3_ as solvent; Figure S3: HMBC NMR spectrum of AS was recorded at 27 °C using CDCl_3_ as solvent; Figure S4: HSQC NMR spectrum of GC was recorded at 27 °C using CDCl_3_ as solvent. The inset shows the HSQC NMR spectrum of GC in the range of 0–8 ppm and 160–190 ppm for ^1^H and APT ^13^C NMR chemical shifts, respectively. No additional correlation signals are observed in this region; Figure S5: COSY NMR spectrum of GC was recorded at 27 °C using CDCl_3_ as solvent; Figure S6: HMBC NMR spectrum of GC was recorded at 27 °C using CDCl_3_ as solvent; Figure S7: HSQC NMR spectrum of D-AS-GC was recorded at 27 °C using CDCl_3_ as solvent. The inset shows the HSQC NMR spectrum of D-AS-GC in the range of 0–8 ppm and 90–170 ppm for ^1^H and APT ^13^C NMR shifts, respectively. No additional correlation signals are observed in this region; Figure S8: ^1^H NMR spectra of (a) propylene glycol, (b) Kolliphor HS 15 and (c) medium-chain triglycerides (MCT) were recorded at 27 °C using D_2_O and CDCl_3_ as solvent; Table S1: HSQC NMR assignment of AS; Table S2: COSY NMR assignment of AS; Table S3: HMBC NMR assignment of AS; Table S4: HSQC NMR assignment of GC; Table S5: COSY NMR assignment of GC; Table S6: HMBC NMR assignment of GC; Table S7: HSQC NMR assignment of D-AS-GC.

## Sample Availability

Samples of the compounds are available from the authors.
