# Supplementary material for: Synthesis and Characterization of Dimeric Artesunate Glycerol Monocaprylate Conjugate and Formulation of Nanoemulsion Preconcentrate
Source: Molecules. 2023 Jul 4;28(13):5208. doi: 10.3390/molecules28135208 (PMC10343907; doi:10.3390/molecules28135208)
Supplement: Supplementary file 1 [file molecules-28-05208-s001.zip › molecules-2470442-supplementary.pdf]

Synthesis and Characterization of Dimeric Artesunate Glycerol Monocaprylate Conjugate and Formulation of Nanoemulsion Preconcentrate

Rana Hore[1], Nazmul Hasan[1], Karsten Mäder[2] and Jörg Kressler[1,*]

[1]Department of Chemistry, Martin Luther University Halle-Wittenberg, Von-Danckelmann-Platz 4, D-06099 Halle/Saale, Germany

[2]Institute of Pharmacy, Martin Luther University Halle-Wittenberg, D-06099, Halle (Saale), Germany

*Correspondence: joerg.kressler@chemie.uni-halle.de

Supplementary Materials

Figure S1. HSQC NMR spectrum of AS was recorded at 27 °C using $CDCl_3$ as solvent. The inset shows the HSQC NMR spectrum of AS in the range of 0–8 ppm and 90–180 ppm for ^1H and APT ^{13}C NMR chemical shifts, respectively. No additional correlation signals are observed in this region.

Figure S2. COSY NMR spectrum of AS was recorded at 27 °C using $CDCl_3$ as solvent.

Figure S3. HMBC NMR spectrum of AS was recorded at 27 °C using $CDCl_3$ as solvent.

Figure S4. HSQC NMR spectrum of GC was recorded at 27 °C using $CDCl_3$ as solvent. The inset shows the HSQC NMR spectrum of GC in the range of 0–8 ppm and 160–190 ppm for ^1H and APT ^{13}C NMR chemical shifts, respectively. No additional correlation signals are observed in this region.

Figure S5. COSY NMR spectrum of GC was recorded at 27 °C using $CDCl_3$ as solvent.

Figure S6. HMBC NMR spectrum of GC was recorded at 27 °C using $CDCl_3$ as solvent.

Figure S7. HSQC NMR spectrum of D-AS-GC was recorded at 27 °C using $CDCl_3$ as solvent. The inset shows the HSQC NMR spectrum of D-AS-GC in the range of 0–8 ppm and 90–170 ppm for ^1H and APT ^{13}C NMR shifts, respectively. No additional correlation signals are observed in this region.

Figure S8. ^1H NMR spectra of (a) propylene glycol, (b) Kolliphor HS 15 and (c) medium-chain triglycerides (MCT) were recorded at 27 °C using D_2O and $CDCl_3$ as solvent.

Table S1. HSQC NMR assignment of AS

Table S2. COSY NMR assignment of AS

Table S3. HMBC NMR assignment of AS

Table S4. HSQC NMR assignment of GC

Table S5. COSY NMR assignment of GC

Table S6. HMBC NMR assignment of GC

Table S7. HSQC NMR assignment of D-AS-GC

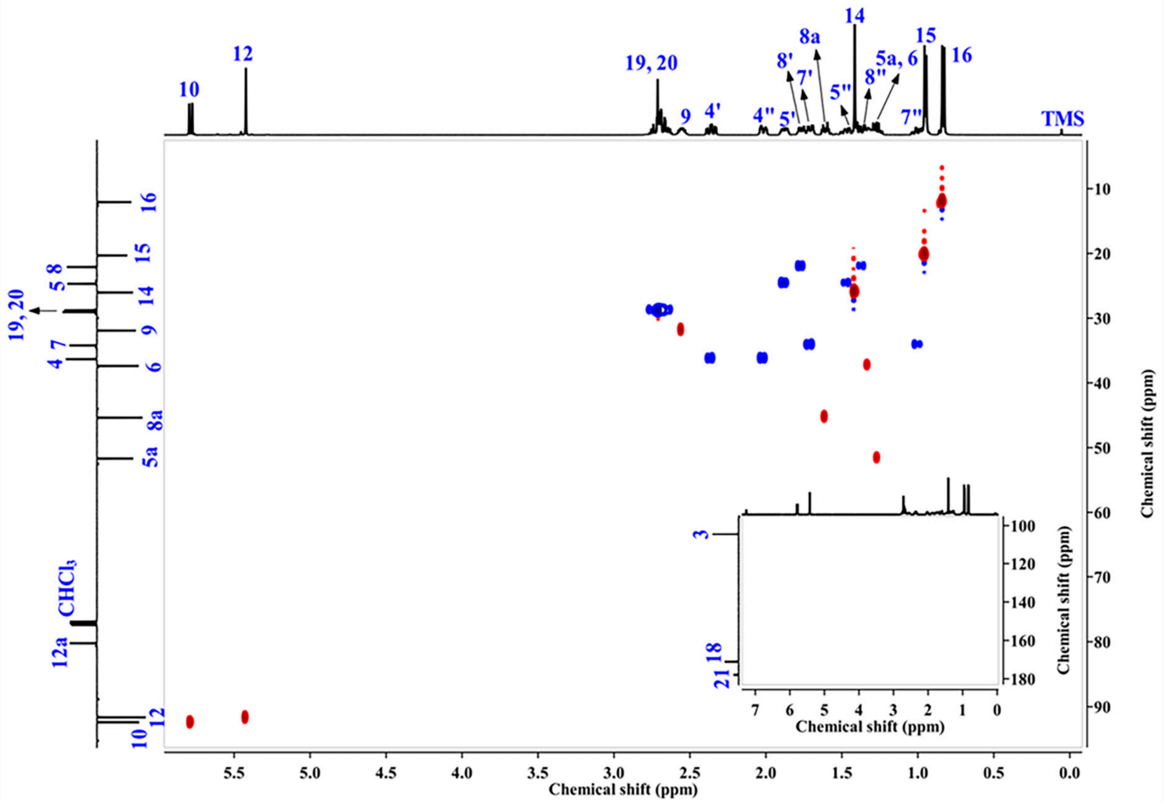

Figure S1. HSQC NMR spectrum of AS was recorded at 27 °C using $CDCl_3$ as solvent. The inset shows the HSQC NMR spectrum of AS in the range of 0–8 ppm and 90–180 ppm for ^1H and APT ^{13}C NMR chemical shifts, respectively. No additional correlation signals are observed in this region.

Table S1. HSQC NMR assignment of AS.

Chemical shift δ_H (ppm)	^1H peak assignment	Chemical shift δ_C (ppm)	APT ^{13}C peak assignment
5.78–5.76	10	92.39	10
5.43–5.40	12	91.63	12
2.73–2.62	19 and 20	29.06–28.79	19 and 20
2.57–2.50	9	31.92	9
2.39–2.33	4'	36.33	4'
2.04–1.99	4''	36.33	4''
1.90–1.86	5'	24.69	5'
1.78	8'	22.10	8'

1.67	7'	34.21	7'
1.62–1.58	8a	45.36	8a
1.50–1.45	5''	24.69	5''
1.42–1.39	14	26.04	14
1.39–1.34	8''	22.10	8''
1.33	6	37.38	6
1.27	5a	51.68	5a
1.04–0.97	7''	34.21	7''
0.96–0.93	15	20.31	15
0.88–0.83	16	12.08	16

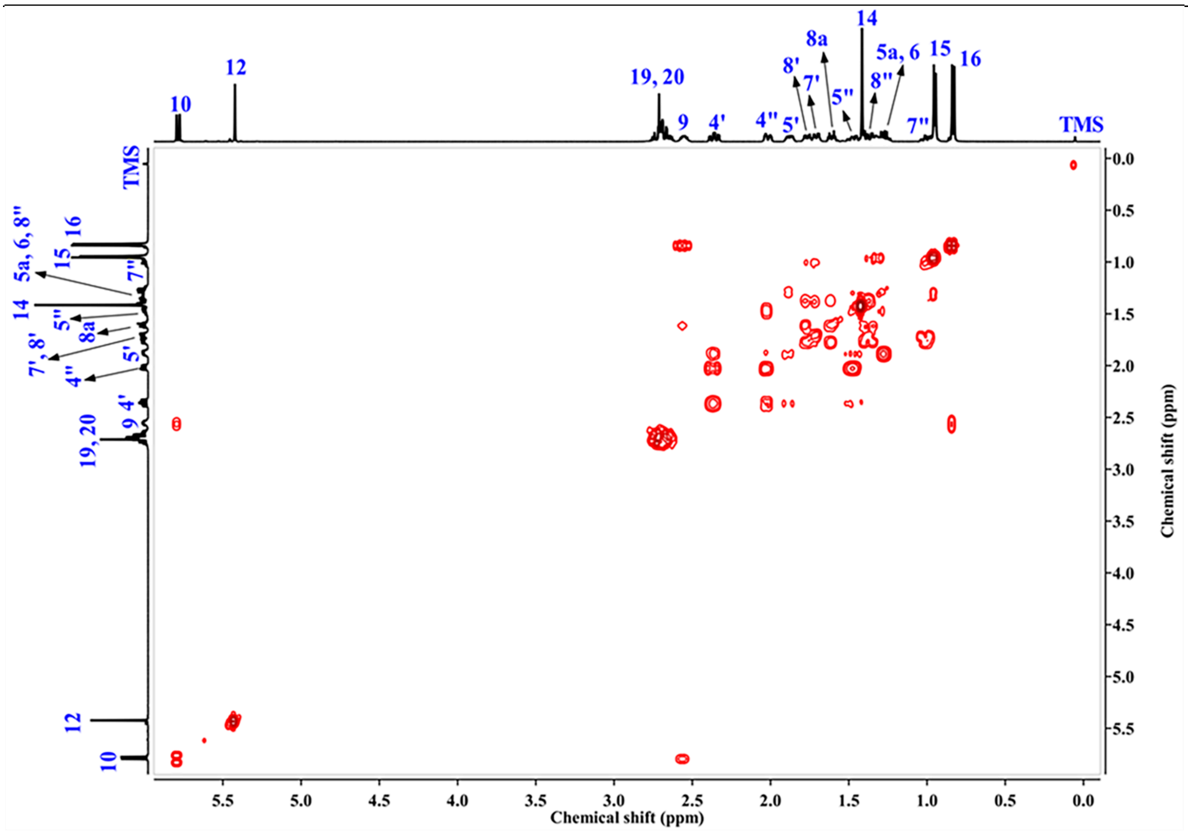

Figure S2. COSY NMR spectrum of AS was recorded at 27 °C using $CDCl_3$ as solvent.

Table S2. COSY NMR assignment of AS.

Chemical shift δ_H (ppm)	Chemical shift δ_H (ppm)	COSY correlation assignment
5.78–5.76	2.57–2.50	10-9
2.57–2.50	0.88-0.83	9-16
2.57–2.50	1.62–1.58	9-8a
2.39–2.33	1.90–1.86	4'-5'

2.39–2.33	2.04–1.99	4'-4''
2.04–1.99	1.50–1.45	4''-5''
2.04–1.99	1.90–1.86	4''-5'
1.90–1.86	1.27	5'-5a
1.78	1.04–0.97	8'-7''
1.67	1.04–0.97	7'-7''
1.67	1.39–1.34	7'-8''
1.78	1.39–1.34	8'-8''
1.78	1.62–1.58	8'-8a
1.62–1.58	1.39–1.34	8a-8''
1.50–1.45	1.27	5''-5a
1.33	0.96–0.93	6-15

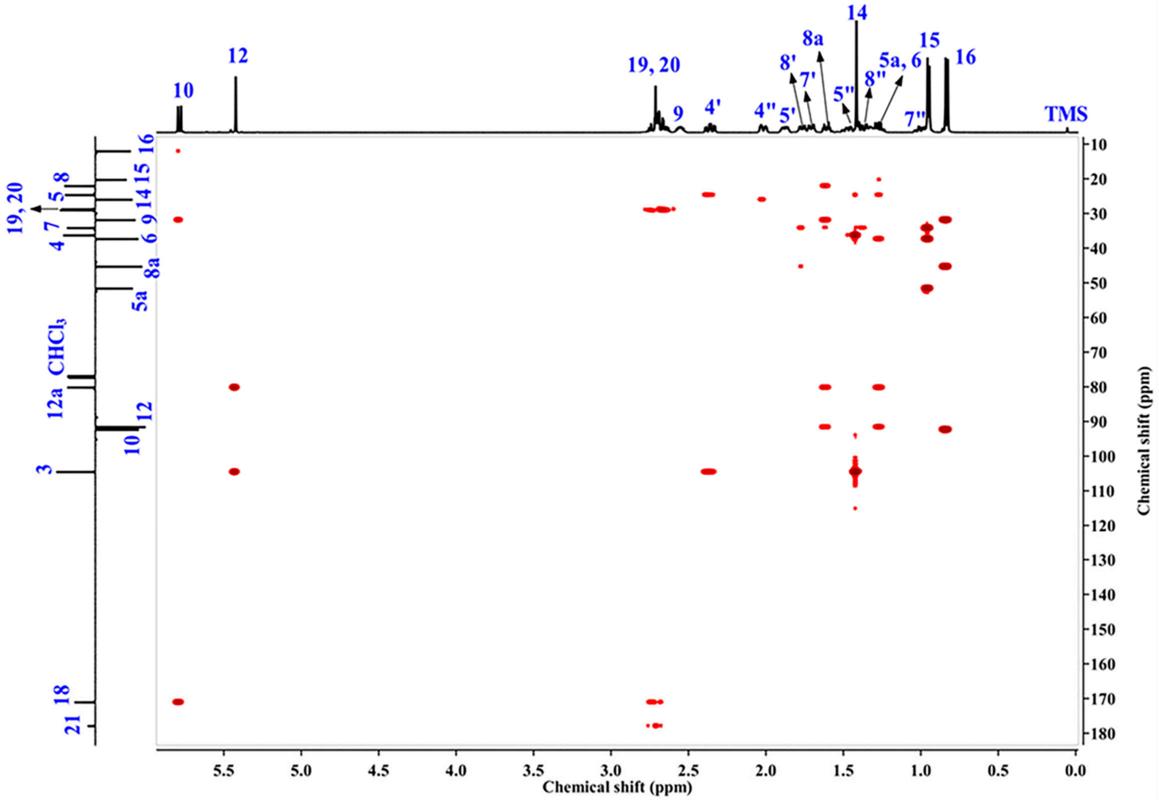

Figure S3. HMBC NMR spectrum of AS was recorded at 27 °C using $CDCl_3$ as solvent.

Table S3. HMBC NMR assignment of AS.

Chemical shift δ_H (ppm)	Chemical shift δ_C (ppm)	HMBC correlation assignment
5.78–5.76	12.08	10-16
5.78–5.76	31.92	10-9
5.78–5.76	171.10	10-18
5.43–5.40	80.22	12-12a

5.43–5.40	104.42	12-3
2.73–2.62	29.06–28.79	19-20 or 20-19
2.73–2.62	171.10	20-18
2.73–2.62	177.94	19-21
2.39–2.33	104.42	4'-3
2.39–2.33	24.69	4'-5
2.04–1.99	26.04	4''-14
1.78	34.21	8'-7
1.78	45.36	8'-8a
1.62–1.58	22.10	8a-8
1.62–1.58	31.92	8a-9
1.62–1.58	80.22	8a-12a
1.62–1.58	91.63	8a-12
1.42–1.39	36.33	14-4
1.42–1.39	104.59	14-3
1.27	24.69	5a-5
1.27	37.38	5a-6
1.27	80.22	5a -12a
1.27	91.63	5a-12
0.96–0.93	34.21	15-7
0.96–0.93	37.38	15-6
0.96–0.93	51.68	15-5a
0.88–0.83	31.92	16-9
0.88–0.83	45.36	16-8a
0.88–0.83	92.39	16-10

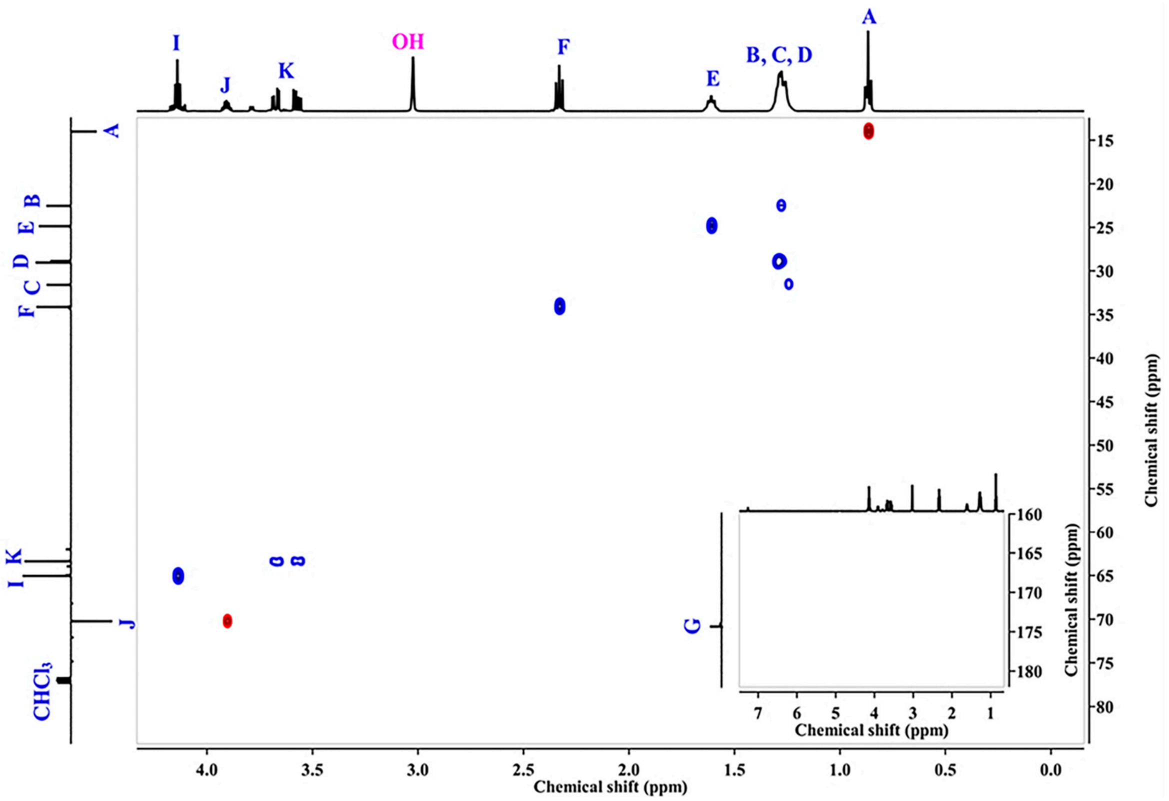

Figure S4. HSQC NMR spectrum of GC was recorded at 27 °C using $CDCl_3$ as solvent. The inset shows the HSQC NMR spectrum of GC in the range of 0–8 ppm and 160–190 ppm for ^{1}H and APT ^{13}C NMR chemical shifts, respectively. No additional correlation signals are observed in this region.

Table S4. HSQC NMR assignment of GC.

Chemical shift δ_H (ppm)	^{1}H peak assignment	Chemical shift δ_C (ppm)	APT ^{13}C peak assignment
4.17–4.10	I	65.20	I
3.92–3.88	J	70.38	J
3.69–3.55	K	63.52	K
2.35–2.31	F	34.27	F
1.64–1.58	E	25.00	E
1.32–1.21	B, C and D	22.69, 31.74 and 29.19	B, C and D
0.88–0.85	A	14.14	A

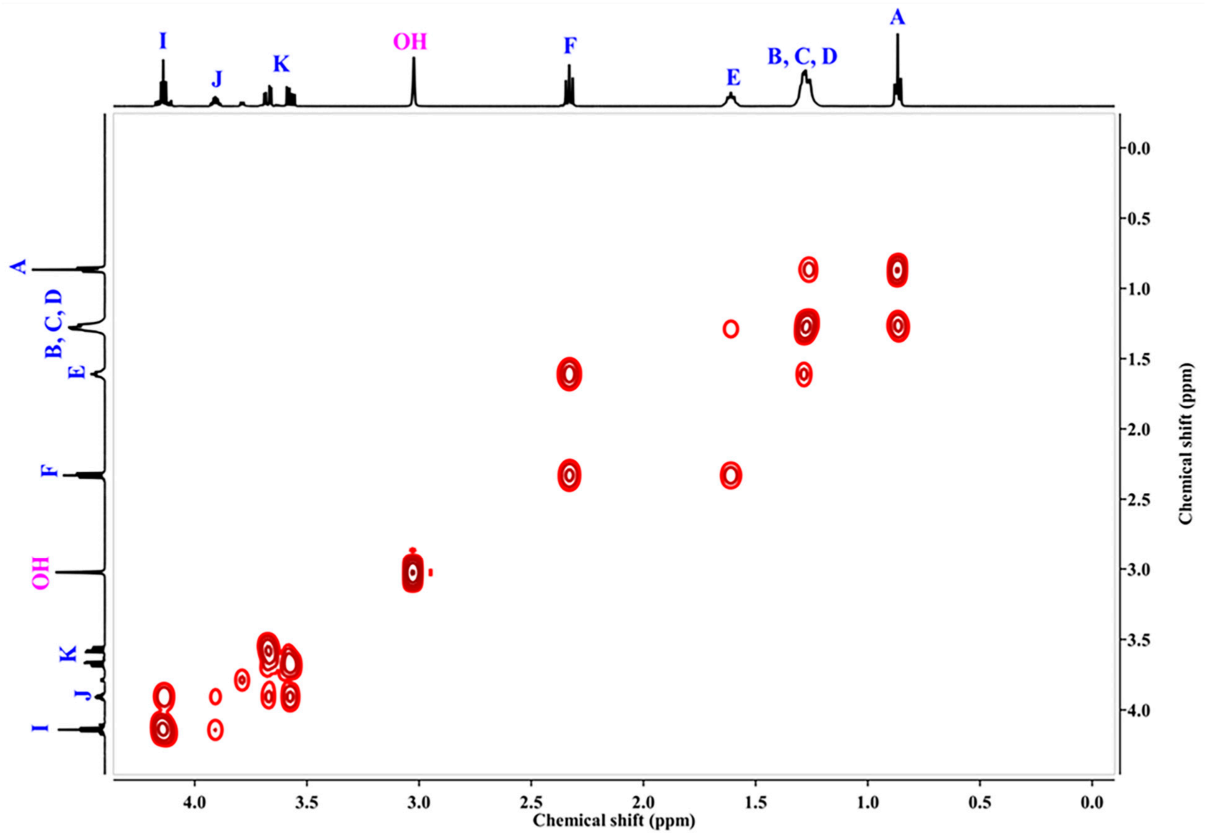

Figure S5. COSY NMR spectrum of GC was recorded at 27 °C using $CDCl_3$ as solvent.

Table S5. COSY NMR assignment of GC.

Chemical shift δ_H (ppm)	Chemical shift δ_H (ppm)	COSY correlation assignment
4.17–4.10	3.92–3.88	I-J
3.69–3.55	3.92–3.88	K-J
2.35–2.31	1.64–1.58	F-E
1.64–1.58	1.32–1.21	E-D
1.32–1.21	0.88–0.85	B-A

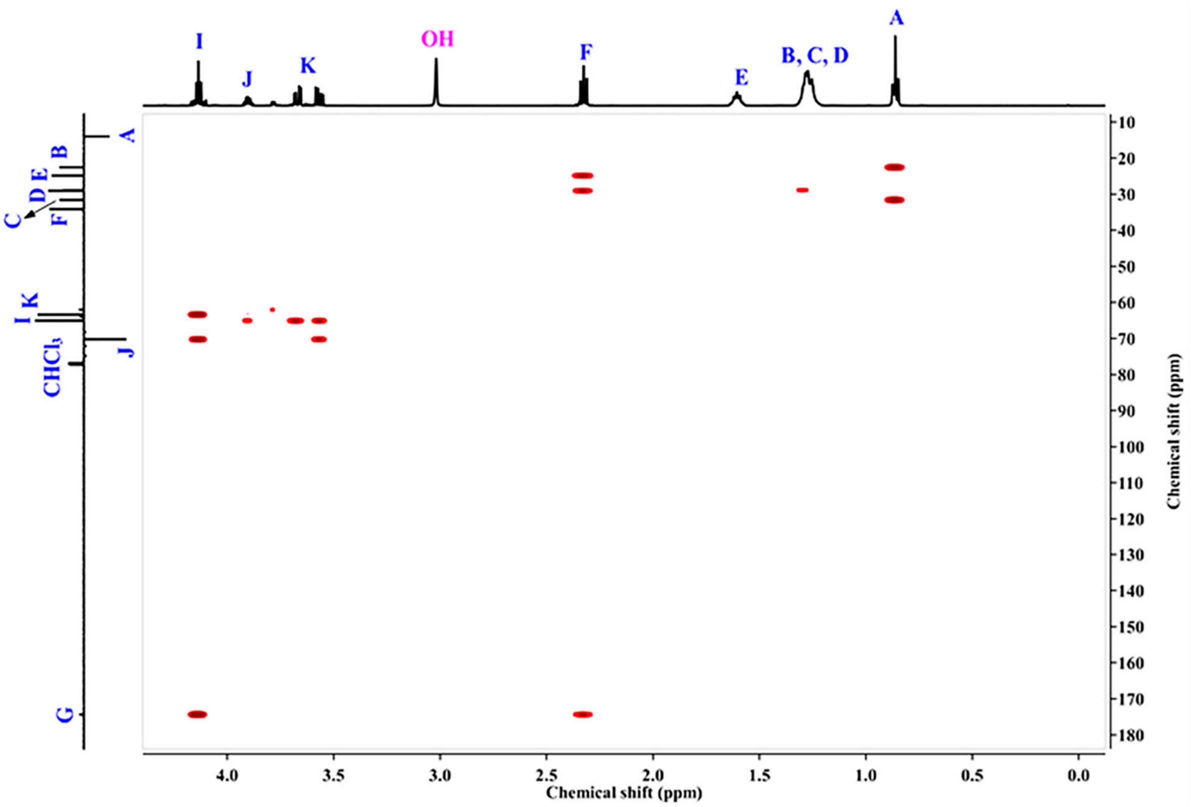

Figure S6. HMBC NMR spectrum of GC was recorded at 27 °C using $CDCl_3$ as solvent.

Table S6. HMBC NMR assignment of GC.

Chemical shift δ_H (ppm)	Chemical shift δ_C (ppm)	HMBC correlation assignment
4.17–4.10	63.52	I-K
4.17–4.10	70.38	I-J
4.17–4.10	174.49	I-G
3.92–3.88	65.20	J-I
3.69–3.55	65.20	K-I
3.69–3.55	70.38	K-J
2.35–2.31	25.00	F-E
2.35–2.31	29.19	F-D
2.35–2.31	174.49	F-G
1.32–1.21	29.19	B/C-D
0.88–0.85	22.69	A-B
0.88–0.85	31.74	A-C

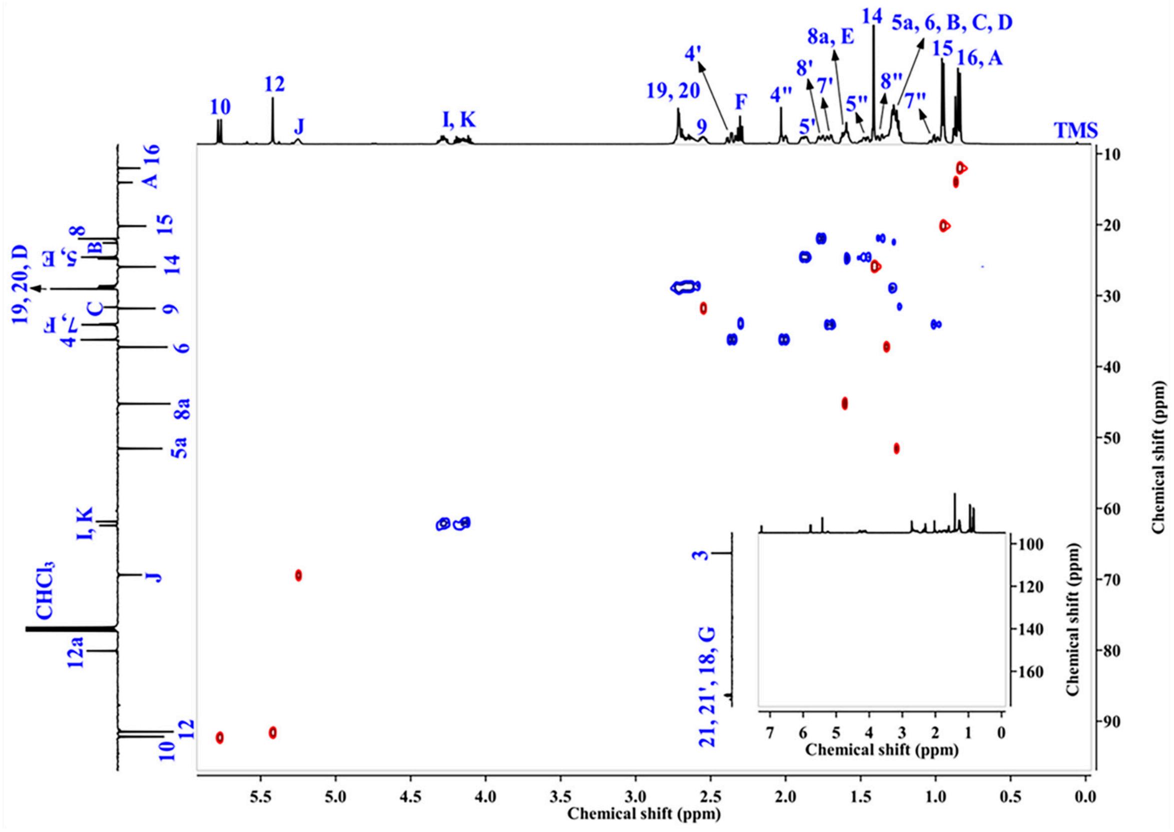

Figure S7. HSQC NMR spectrum of D-AS-GC was recorded at 27 °C using $CDCl_3$ as solvent. The inset shows the HSQC NMR spectrum of D-AS-GC in the range of 0–8 ppm and 90–170 ppm for ^{1}H and APT ^{13}C NMR shifts, respectively. No additional correlation signals are observed in this region.

Table S7. HSQC NMR assignment of D-AS-GC.

Chemical shift δ_H (ppm)	^{1}H peak assignment	Chemical shift δ_C (ppm)	APT ^{13}C peak assignment
5.78–5.76	10	92.19	10
5.43–5.40	12	91.48	12
5.28–5.23	J	69.37	J
4.32–4.09	I, K	62.41–61.86	I, K
2.73–2.62	19 and 20	29.04–28.62	19 and 20
2.57–2.50	9	31.80	9
2.39–2.29	4'	36.22	4
2.39–2.29	F	34.10–33.98	F
2.04–1.99	4"	36.22	4
1.90–1.86	5'	24.84–24.58	5
1.78–1.69	8'	21.98	8

1.78–1.69	7'	34.10–33.98	7
1.62–1.58	E	24.84–24.58	E
1.62–1.58	8a	45.24	8a
1.50–1.45	5''	24.84–24.58	5
1.42–1.39	14	25.93	14
1.39–1.34	8''	21.98	8
1.31–1.23	6	37.25	6
1.31–1.23	B	22.51	B
1.31–1.23	D	31.63	C
1.31–1.23	C	29.04–28.62	D
1.31–1.23	5a	51.57	5a
1.04–0.97	7''	34.10–33.98	7
0.96–0.93	15	20.19	15
0.88–0.83	A	14.05	A
0.88–0.83	16	12.03	16

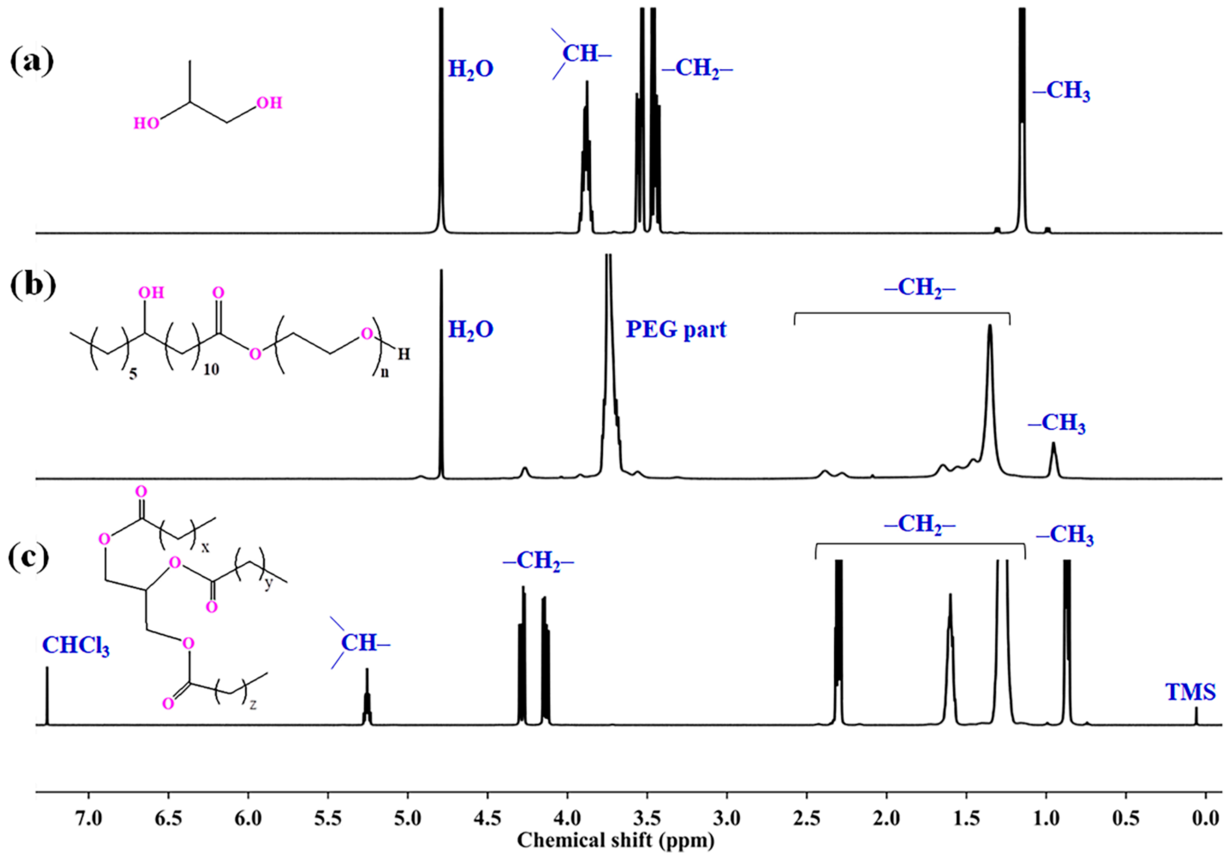

Figure S8. ^{1}H NMR spectra of (a) propylene glycol, (b) Kolliphor HS 15 and (c) medium-chain triglyceride (MCT) were recorded at 27 °C using D_2O and $CDCl_3$ as solvent.
